# Development and Validation of One-Step Reverse Transcription-Droplet Digital PCR for Plum Pox Virus Detection and Quantification from Plant Purified RNA and Crude Extract

**DOI:** 10.3390/plants13233276

**Published:** 2024-11-22

**Authors:** Giorgia Bertinelli, Lorenza Tizzani, Marta Luigi, Simona Monticelli, Vincenza Ilardi

**Affiliations:** 1 CREA Research Centre for Plant Protection and Certification, Via C.G. Bertero 22, 00156 Rome, Italy; giorgia.bertinelli@crea.gov.it (G.B.); lorenza.tizzani@crea.gov.it (L.T.); marta.luigi@crea.gov.it (M.L.); 2 CREA Research Centre for Olive, Fruit and Citrus Crops, Via di Fioranello 52, 00134 Rome, Italy; simona.monticelli@crea.gov.it

**Keywords:** plum pox virus, sharka, *Prunus* spp., real-time PCR, RT-ddPCR, PPV strains

## Abstract

Plum pox virus (PPV) is the etiological agent of sharka, the most important viral disease of stone fruit worldwide. In this study, a one-step reverse transcription real-time PCR test (RT-qPCR) was modified and translated as a one-step RT-droplet digital PCR (RT-ddPCR) for sensitive, direct, and accurate detection and quantification of PPV. The modified RT-qPCR and RT-ddPCR PPV detection tests were validated using both plant purified total RNA (TRNA) and crude extract as templates. The proposed tests were sensitive, specific, selective, repeatable, and reproducible in detecting PPV from fresh, lyophilized, and *in vitro* plant samples. RT-ddPCR was more sensitive than RT-qPCR in detecting PPV using purified TRNA while showing the same sensitivity using crude extract. This work highlights the robustness, time-saving, and cost-effective nature of the proposed one-step RT-ddPCR test, offering a potential reduction in resources for PPV detection and quantification even with raw extracts.

## 1. Introduction

*Plum pox virus* (PPV), a member of the Potyvirus genus, is the causal agent of sharka, the most devastating viral disease of stone fruits regarding agronomic impact and economic importance [1]. PPV is considered one of the “top ten” viruses in molecular plant pathology [2], and it is categorized as a quarantine pest in many countries (https://gd.eppo.int/taxon/PPV000/categorization, accessed on 12 June 2024). 

The PPV genome is a single strand of positive-sense RNA of about 9770 nucleotides in length, encapsidated by a single type of coat protein (CP) in a flexuous rod-shaped particle. Currently, a total of ten PPV genetic strains are recognized: Dideron (D), Marcus (M), El Amar (EA), Cherry (C), Recombinant (Rec), Turkish (T), Winona (W), Ancestral (An), Cherry Russian (CR), and Cherry Volga (CV) [3,4,5]. The main strains with an extensive geographical distribution and economic impact are PPV-M, D, and Rec [5,6].

Despite several efforts to eradicate PPV infection or contain its spread, the virus is found worldwide in several important prunus-producing areas [7,8,9,10,11].

It is essential to have fast, sensitive, and quantitative detection methods for studies aimed at obtaining PPV resistance and containing its spread. Several molecular methods were developed to detect PPV, each having its own peculiarity. For example, reverse transcription loop-mediated isothermal amplification (RT-LAMP) is a fast and cheap method even under field conditions (e.g., [12,13]). According to the International Plant Protection Convention [1], most sensitive and effective PPV detection methods use reverse transcription quantitative real-time PCR (RT-qPCR) [14,15]. In particular, Olmos and coauthors [15] developed a Taqman RT-qPCR assay that targets PPV-D and PPV-M using strain-specific reverse primers. This assay is compatible with a fast extraction protocol based on direct sample preparation [16]. 

However, for sample quantification, qPCR has the limitation of needing a known standard [17]. Indeed, quickly quantifying the virus in the plant is essential in many studies, such as those for developing virus control systems, during breeding programs for obtaining resistance, in plant–virus interaction studies, and to standardized reference materials for official analyses. In the past few years, digital PCR (dPCR) has been introduced as a new diagnostic tool [18]. Digital-PCR-based methods, such as droplet digital PCR (ddPCR) and chip-based digital PCR, have already been shown to have the potential to improve the limitations of qPCR, such as the absolute concentration of the target copies in the initial sample [19,20]. Moreover, ddPCR shows tolerance to PCR inhibitors in plant and soil samples [21] and has shown high specificity and a low limit of detection (LOD) across many applications. General reporting on sensitivity in studies using qPCR and ddPCR methods is that the limit of detection of ddPCR can be 10–100 times more sensitive than qPCR. For the above reasons, ddPCR and reverse transcription ddPCR (RT-ddPCR) are spreading among plant virology as valid and sensible detection and quantification techniques [22,23,24,25,26,27,28,29,30,31,32]. However, the cost-effectiveness of ddPCR is one of its main issues. It is reported that ddPCR costs two times as much as qPCR, and its lesser availability has prevented the distribution of ddPCR technology worldwide [33]. However, especially when it comes to certification of plant asymptomatic material, it is essential to have a sensitive detection method, and this is the reason why the ddPCR protocol was proposed to be applied for use in national certification plant programs to prevent the importation of infected nursery stock [34]. 

For the molecular detection/quantification of the virus, the samples must be processed to purify the nucleic acids. Commercial kits are usually preferred to avoid using organic solvents, speed up the extraction, and standardize the method as much as possible among laboratories [35]. Direct sample preparation, circumventing the RNA purification step, was implemented to further speed up and economize the test. This consists of using crude plant extract spotted on a membrane as a template [36,37]. 

This work aimed to develop a validated, fast, sensitive, and affordable one-step RT-droplet digital PCR for PPV detection and quantification from plant RNA and crude extract.

## 2. Results and Discussion

In the present study, the one-step RT-qPCR test developed by Olmos et al. (2005) [15] and modified as reported in Pasquini et al. [16] and PM 7/32 (2) [11] was further simplified to use a single reverse primer selected in a more conserved *CP* region able to identify the most important PPV strains (i.e., D, M, and Rec). This new test was then transposed to RT-ddPCR to obtain a one-step test for detecting and quantifying PPV strains. The reverse transcription enzyme was added to the ddPCR mix to make the method cheaper than a commercial one-step RT-ddPCR mix, reducing the costs by about a third. Costs and time were also reduced using crude plant extract immobilized on a nylon membrane. The performance of the RT-qPCR and RT-ddPCR was validated through the EPPO standard PM 7/98 (5) [38] using both TRNAs and crude extracts of PPV-infected plants (fresh *in vivo* leaf and wood, lyophilized leaf, and *in vitro* shoot) as templates.

### 2.1. Reverse Primer Design and RT-qPCR Development

A single reverse PCR primer (PPVrUn-R 5′-GGAGGTTGTGCATGTTGCGATT-3′), to be used instead of P316 M and P316 D primers [15], was designed on a PPV *CP* gene region conserved among the PPV strains and overlapping the four conserved nucleotides at the 3′ end of the P316 M and P316 D primers. The expected amplicon, using PPVrUn-R coupled with the forward primer P241 by Olmos and colleagues [15], was 94 nt in length. PPVrUn-R primer was designed and tested *in silico* with 485 nucleotide sequences representing the genetic variability of the PPV *CP* (sequences of GenBank and CREA-DC PPV collection: [39] and those obtained in this work; accession numbers: OL771187, OL771188, and OL771189). PPVrUn-R primer was highly homologous to all PPV strains. Indeed, the *in silico* analysis highlighted a complete identity with 311 out of 313 PPV-D, 66 out of 68 PPV-M, and all the PPV-Rec, T, EA, C, and SC tested. There was a difference in a single nucleotide (T instead of C) for the W strain. The forward primer and probe derived from Olmos and colleagues [15] were also subjected to *in silico* testing, and their pattern of identity was consistent with those observed for the PPVrUn-R primer (Supplementary Table S1). The *in vitro* efficiency of the new oligonucleotide set was then compared with the Olmos one in RT-qPCR. Comparable results were obtained in amplifying the PPV *CP* region both from purified TRNA and crude extract of PPV-D- and PPV-M-infected plants from the CREA-DC collection (six PPV-D isolates for TRNA, three isolates for crude extracts, and one PPV-M isolate for both) (Table 1 and Supplementary Figure S1).

### 2.2. RT-ddPCR Development

**Figure 1:**
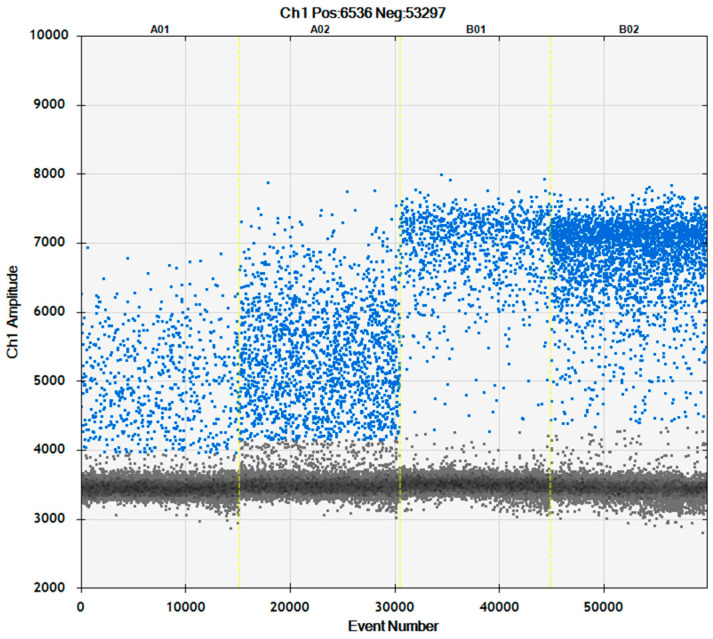
The impact of the RNAse inhibitor on RT-ddPCR. A01–A02 without and B01–B02 with the RNase inhibitor. A01 and B01—CREA-DC-PPV6 (PPV-M). A02 and B02—CREA-DC-PPV7 (PPV-D). Blue dots—positive droplets with target amplification. Gray dots—negative droplets without amplification. The threshold for the droplet positivity was set automatically by QuantaSoft software version 1.7.4.

Transposing a test from RT-qPCR to RT-ddPCR requires optimizing parameters that may influence the reaction and, consequently, the amplitude and clustering of the droplets. To improve the reverse transcription process, the effect of an RNase inhibitor was assessed. Adding an RNase inhibitor to the RT-ddPCR mix improved the separation of the clusters of positive and negative droplets (Figure 1). This finding is probably because in digital PCR, it is possible to distinguish between different amplification products in the same reaction as the compartmentalization allows such a distinction [40]. Therefore, without an RNase inhibitor in the reaction mixture, the target RNA is partially degraded, producing an additional population of droplets with fluorescence values defined as “rain”. Hence, an RNase inhibitor was used in all subsequent experiments.

To further optimize the reverse transcription process, the temperatures of 45 and 48 °C were compared. Although both temperatures gave good droplet separation, 45 °C resulted in a better quantification of the target (Supplementary Figure S2).

As an end-point PCR assay, ddPCR can be improved by increasing the number of cycles. Usually, 40 cycles is enough to reach an optimal amplification [26,28,29], but in some cases, the number of cycles can be increased to 45 [25,41,42]. In this study, a preliminary investigation test suggested that increasing the reaction to 45 cycles allowed a better droplet separation in RT-ddPCR (Supplementary Figure S3).

**Figure 2:**
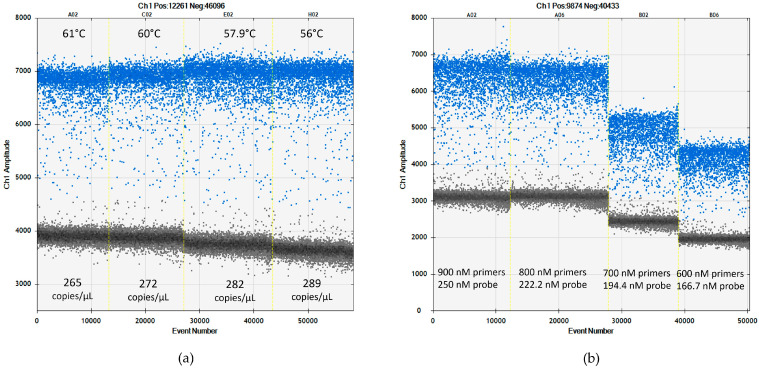
Influence of annealing temperature and primers/probe concentration on separation of positive (blue) and negative (gray) droplets using CREA-DC-PPV7 isolate. (**a**) The best resolution and highest number of target copies/µL were obtained at an annealing temperature of 56 °C (column H02). (**b**) The best resolution was observed with the combination of 800 nM primers and a 222.2 nM probe (column A06). The threshold for the droplet positivity was set by QuantaSoft software version 1.7.4.

The annealing temperature was also assessed, ranging from 56 to 61 °C using M (CREA-DC-PPV6) and D (CREA-DC-PPV7) PPV strains as targets. Four different temperatures were compared, 56, 57.9, 60, and 61 °C, taking into consideration that the temperature in qPCR was 60 °C and that changing it to lower values may increase in digital resolution [40]. Although all the tested temperatures showed good resolution between clusters of positive and negative droplets, 56 °C resulted in better quantification and droplet separation (Figure 2a). This temperature is 4 °C lower than that used in RT-qPCR. The same result was obtained by transferring an RT-qPCR assay to an RT-ddPCR for other potyviruses (i.e., potato virus Y [22], watermelon mosaic virus, and zucchini yellow mosaic virus [30]). These data indicate the importance of fine-tuning the annealing temperature parameter moving from RT-qPCR to RT-ddPCR.

To define the best primers/probe concentration, CREA-DC-PPV6 and CREA-DC-PPV7 isolates were used. The range was from 900 nM/250 nM to 600 nM/166.7 nM, maintaining the recommended ratio of primers/probe 3.6 [43]. The optimal condition observed was 800 nM primers and a 222.2 nM probe (Figure 2b).

### 2.3. Validation of One-Step RT-qPCR and RT-ddPCR

#### 2.3.1. Analytical Specificity

Primers and probe inclusivity were tested *in silico* as described in the above paragraph “Reverse primer design and RT-qPCR development”.

The *in silico* exclusivity was tested against the nucleotide sequences of viruses and viroids hosted by *Prunus* spp. indicated in the EPPO standard certification scheme PM 4/30 (1) [44], and no significant matches were found, confirming that the oligonucleotide set was specific to plum pox virus (Supplementary Table S2).

The inclusivity of RT-qPCR and RT-ddPCR was also evaluated *in vitro* using TRNAs and crude extracts as templates. All five PPV strains (Table 1) were successfully detected by both tests (Supplementary Figure S4; Supplementary Table S2), confirming that the PPVrUn-R reverse primer was useful for wide-range PPV detection. This result is consistent with those obtained by the Olmos et al. RT-qPCR test [15] in a test performance study performed in the framework of the EU-funded VALITEST project (https://www.valitest.eu/) and reported in the EPPO Standard PM 7/32 (2) [11].

The exclusivity *in vitro* test, performed on the TRNA of six viruses and two viroids associated with *Prunus* spp. (Table 1), gave no positive results (Supplementary Table S2). The exclusivity test, performed on the crude extracts of three major *Prunus*-related viruses, confirmed the absence of cross-reactions also using this kind of raw template (Supplementary Table S2).

#### 2.3.2. Analytical Sensitivity

**Figure 3:**
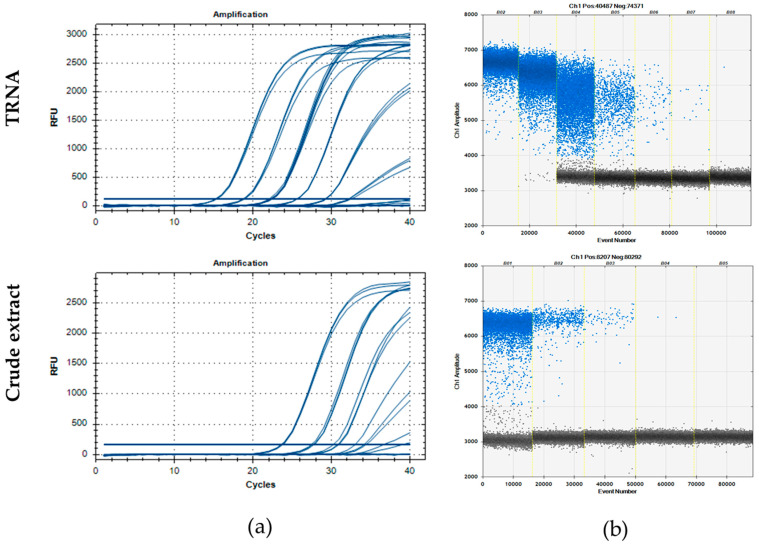
Analytical sensitivity of CREA-DC-PPV Rec BR TRNA (upper panels) and crude extract (lower panels) in RT-qPCR (**a**) and RT-ddPCR (**b**). The undiluted TRNA and the first 1:10 dilution were not quantifiable in ddPCR because all droplets were positive (upper panel, columns B02-B03).

The analytical sensitivity was assessed using the isolates CREA-DC-PPV6, CREA-DC-PPV10, and CREA-DC-PPV Rec BR (Table 2), which belong to the most agronomically important PPV strains (M, D, and Rec, respectively). The limit of detection (LOD) of TRNAs corresponded to a 10^−5^ dilution for RT-qPCR and 10^−6^ for RT-ddPCR for all the analyzed PPV isolates. The LOD using crude extracts was 10^−3^ for CREA-DC-PPV Rec BR and CREA-DC-PPV6, and 10^−2^ for CREA-DC-PPV10 for both tests, although the standard deviations of the results obtained by RT-qPCR were always higher than those of RT-ddPCR (Figure 3 and Table 2).

The correlation coefficient (R^2^) obtained by linear regression analysis showed good linearity of the amplification for both RT-qPCR and RT-ddPCR (R^2^ > 0.99). For TRNAs, RT-qPCR efficiency was in the optimal range (102–105%), while for crude extracts, it decreased (80–98%). This result was expected, as the crude extract is obtained without purification steps and has a more considerable concentration of inhibitors. If not diluted in water at the end of the protocol, the surfactant (Triton X-100) used for the crude extraction method interferes with droplet generation, resulting in an unacceptable number of droplets during the RT-ddPCR analysis. In the case of TRNAs, dilution up to 10^−1^ resulted in RT-ddPCR saturation as no negative droplets were detected (Figure 3). The reported result is in line with those reported by other studies [22,23,24,25,26,30,32,45], which further validates our findings.

Comparisons between the assays showed that RT-ddPCR was ten times more sensitive than RT-qPCR when using TRNAs of PPV-infected plants. This result agrees with those obtained by Mehle and colleagues for potato virus Y [22]. The same detection limit was observed using crude extracts (Table 2). This last result seems to disagree with the assumption that ddPCR is less affected by inhibitors (e.g., [39]). However, it should be considered that, in the crude extracts, plant debris could influence the formation of the droplets and/or the performance of the end-point PCR inside them. ddPCR found its strength in partitioning; the sample is divided into millions of partitions by an oil emulsion, generating droplets. In this context, the presence of plant debris can affect the possibility of good sample partitions in the generated droplets, thus affecting the sensitivity of the reaction.

#### 2.3.3. Selectivity, Repeatability, and Reproducibility

The RT-qPCR and RT-ddPCR methods consistently quantified PPV, regardless of the Prunus-related matrix (Table 3). Even with woody samples and senescent and/or asymptomatic leaves, both tests successfully detected PPV (Supplementary Figure S5). This successful detection in wood or senescent leaves makes the developed RT-ddPCR test usable throughout the year, a characteristic that has always been sought after [46]. The successful application of both detection tests to *in vitro* cultured shoots as a matrix (Supplementary Figure S6) is a significant advantage. This capability could be crucial for *in vitro* experiments to produce virus-free plants [47] or conduct contained resistance assays with genetically modified plants and quarantine viruses [48,49].

Validation also involves the evaluation of the test’s performance in terms of repeatability (the level of agreement between replicates of a sample tested under the same conditions) and reproducibility (the ability of a test to provide consistent results when applied to aliquots of the same sample tested under different conditions, such as time, people, equipment, and location) [38]. The repeatability and reproducibility analysis performed using both TRNAs and crude extracts of the three isolates CREA-DC-PPV Rec BR (PPV-Rec strain), CREA-DC-PPV10 (PPV-D strain), and CREA-DC-PPV6 (PPV-M strain) demonstrated that both RT-qPCR (Supplementary Table S3) and RT-ddPCR (Supplementary Table S4) were 100% repeatable and reproducible.

## 3. Materials and Methods

### 3.1. Viruses, Viroids, and Plant Materials

The virus and viroid isolates used in this study are described in Table 1. PPV isolates were maintained under greenhouse conditions in the peach GF305 and/or *Nicotiana benthamiana*. Fresh and/or lyophilized infected leaf samples were tested. For CREA-DC-PPV6, CREA-DC-PPV7, and CREA-DC-PPV10, a woody infected matrix was also analyzed. GF305 shoots were cultured *in vitro* from branches of greenhouse-grown CREA-DC-PPV6- and CREA-DC-PPV9-infected and healthy plants, as described in the next paragraph.

### 3.2. In Vitro GF305 Shoots’ Establishment

Branches collected in spring from greenhouse-grown plants were cut into uninodal segments and washed with water and detergent (Lysoform^®^ medical soap, Unilever Italia SpA, Rome, Italy) for one hour under agitation. The segments were then rinsed under tap water and subjected to a disinfection treatment, consisting of 5 min immersion in ethanol 70%, 15 min in NaClO 1%, a further 5 min in ethanol 70%, and 15 min in NaClO 0.5%, after rinsing in sterile distilled water at each change of solution. After disinfection, the meristematic dome and a few leaf primordia were dissected from lateral and apical buds of nodal segments, removing the bark surrounding the buds. Explants 1–2 mm long were transferred on a culture medium, following the method of Damiano and coauthors [50]. Explants were cultured onto culture media and maintained at 24 ± 2 °C under cool, white, fluorescent light (37.5 µmol m^−2^ s^−1^ of photosynthetically active photon flux) with a 16 h photoperiod. Proliferating shoots were transferred to fresh media every 15 days.

### 3.3. Sample Preparation

Total RNA (TRNA) was extracted from fresh *in vivo* plant material (both leaves and bark), *in vitro* leaves, and lyophilized leaf samples using RNeasy Plant Mini Kit (Qiagen, Hilden, Germany), following the manufacturer’s instructions. The samples were ground into liquid nitrogen before extraction. A healthy control was always included among the samples to monitor for potential contamination during the extraction procedure. The final elution was repeated twice, followed by 1:10 sample dilution in RNase-free water. The RNA was stored at −80 °C for further procedures. 

Crude extracts were obtained, as reported by Capote and colleagues [16,36]. Briefly, fresh leaf samples were placed in plastic bags and ground 1/20 (*w*/*v*) with extraction buffer (PBS buffer, pH 7.2, supplemented with 2% (*w*/*v*) PVP and 0.2% (*w*/*v*) sodium diethyl dithiocarbamate). Then, 5 µL of homogenate was spotted on a nylon membrane placed in a 1.5 mL tube, and 100 µL of 0.5% Triton X-100 was added. The crude extracts were stored at −20 °C until use.

### 3.4. Design of the Reverse Primer

A single PPV *CP* reverse PCR primer was designed using complete PPV genome sequences (retrieved from the Nucleotide GenBank database, National Center for Biotechnology Information (NCBI)), which were then trimmed to obtain the *CP* sequence using Galaxy Europe software version 2.31.1 [51]. The obtained *CP* sequences were aligned using Clustal Omega [52]. The *in silico* exclusivity of the primer was tested using the BLAST^®^ tool (NCBI). The list of the pathogens used for *in silico* exclusivity is reported in Supplementary Table S2.

### 3.5. PPV CP Gene Cloning and Sequencing

The PPV *CP* gene was cloned from the three PPV isolates, CREA-DC-PPV6, CREA-DC-PPV7, and CREA-DC-PPV10 (Table 1). Primers for cloning were designed in this study. The reverse primer (PPV-Un_CPR: 5′-TTATGATAGATACCGAGACCAC-3′) was homologous to both D and M strains, while the two different forward primers were strain-specific and targeted PPV-D and PPV-M, respectively (PPV-D_CPF: 5′-CGACGACATTAACGATGATGG-3′; PPV-M_CPF: 5′-CGGAAATTGAGAGATACCTCG -3′). The expected amplicon length for PPV-D and PPV-M was 1060 and 1091 nt, respectively.

### 3.6. RT-qPCR

For the RT-qPCR assays, the TaqMan^®^ RNA-to-Ct™ 1-Step Kit (Applied Biosystems, Foster City, CA, USA) was used. Primers and probe concentration were as in Pasquini and coauthors [16]. The final reaction volume was 25 µL and contained 1X master mix, 1X RT enzyme mix, 1 µM primer P241 [15], 1 µM primer PPVrUn-R (designed in this study), 150 nM Taqman probe PPV-DM [15], 2 µL of RNA sample, and RNase-free water to reach the final volume. Thermal cycling conditions were 48 °C for 30 min, 10 min at 95 °C, 40 cycles of 15 s at 95 °C, and 1 min at 60 °C. The reaction was conducted in a CFX96 Touch Real-Time PCR Detection System (Bio-Rad, Hercules, CA, USA), with at least two replicates per sample. The reaction was considered positive if it produced an exponential amplification curve. In the positive results, quantification cycles (Cq) were determined using CFX Maestro™ Software (Bio-Rad).

### 3.7. RT-ddPCR

For the RT-ddPCR reaction, ddPCR Supermix for Probes (No dUTP) (Bio-Rad,) was used. The final reaction volume was 20 µL and contained 1X supermix, 10 mM dithiothreitol (DTT), 40U RNase out (Thermo Fisher Scientific, Waltham, MA, USA), 20U M-MLV Reverse Transcriptase (Thermo Fisher Scientific), 800 nM primers (P241 and PPVrUn), 222.2 nM probe (PPV-DM), 2 µL of RNA sample, and RNase-free water to reach the final volume. Reaction mixtures and droplet generation oil for probes were loaded in a DG8 Cartridge, and droplets were generated by placing the cartridge in a QX200 Droplet Generator (Bio-Rad). The droplets were transferred to a 96-well ddPCR plate, which was sealed with a pierceable foil heat seal in a PX1 Plate Sealer (Bio-Rad). The amplification was performed in a T100 thermal cycler (Bio-Rad). Thermal cycling conditions were as follows: 60 min reverse transcription at 45 °C, 10 min at 95 °C, 45 cycles of 30 s at 94 °C and 1 min at 56 °C, 10 min final enzyme deactivation at 98 °C, and a final cooling step of 40 min at 4 °C to stabilize the droplets. All steps had a ramp rate of 2 °C/s. After the amplification, the plate was transferred to a QX200 Droplet Reader (Bio-Rad). 

Positive droplets containing amplified products were discriminated from the negatives by applying a fluorescence amplitude threshold set on the negative controls, using QuantaSoft software version 1.7.4 (Bio-Rad). Data generated were reected if <10,000 droplets were analyzed per 20 µL of reaction or if >99.99% of the droplets were positive.

### 3.8. Validation Experiments

Both RT-qPCR and RT-ddPCR tests were validated according to the European and Mediterranean Plant Protection Organization (EPPO) standard PM 7/98 (5) [38].

For the analytical specificity, the inclusivity was tested using eight PPV isolates belonging to five PPV strains: PPV-M (three isolates), PPV-D (six isolates), Rec, EA, and SwC. For exclusivity, 11 isolates belonging to seven viruses and two viroids were tested, including those indicated in the EPPO standard certification scheme of *Prunus* spp. (PM 4/30 (1) [44]) (Table 1; Supplementary Table S2). *In silico* comparison of primer/probe sequences to sequences in the NCBI genomic library Nucleotide GenBank database was performed using BLAST ^®^ (NCBI).

Analytical sensitivity, repeatability, and reproducibility were tested using the isolates CREA-DC-PPV Rec BR (PPV-Rec), CREA-DC-PPV6 (PPV-M), and CREA-DC-PPV10 (PPV-D). Analytical sensitivity was assessed by preparing 10-fold serial dilutions of PPV-infected samples of TRNA or crude extracts in a healthy sample matrix. Each sample was tested in technical duplicate or triplicate. To minimize variability and compare RT-qPCR with RT-ddPCR, the same sample dilutions were analyzed with both methods on the same day, and the limit of detection (LOD) was assessed for both tests.

To compare the sensitivity of the techniques, Cq values from RT-qPCR were converted to copy numbers using the RT-ddPCR absolute quantification as a calibrator. Cq values and copy numbers of the sample dilutions were interpolated, and linear regression was used to obtain the equations to calculate the copy number in RT-qPCR, as in Mehle and colleagues [22].

To test selectivity, seven different *Prunus* species were used (*P. persica*, *P. armeniaca*, *P. domestica*, *P. salicina*, *P. cerasifera*, *P. avium*, and *P. amygdalus*). The TRNA of each matrix was mixed 1:2 (*v*/*v*) with the TRNA of the GF305 plant infected with CREA-DC-PPV6 isolate and tested in triplicate for RT-qPCR and in duplicate for RT-ddPCR.

Repeatability was assessed by analyzing three LOD concentration replicates of the same isolates used for analytical sensitivity assessment.

For reproducibility, the same samples were analyzed six days later by a different operator in a different laboratory.

## 4. Conclusions

This study was intended to develop a quick and cost-effective one-step RT-ddPCR method for PPV detection and quantification. A one-step RT-qPCR test [15] was modified and transposed to RT-ddPCR using TRNA and crude extract templates. Both tests were validated following EPPO standard PM 7/98 (5) [38]. The tests were specific for the most agronomically relevant PPV strains (M, D, and Rec) and other genetically distantly related strains (EA and SwC). Using TRNAs, RT-ddPCR was 10 times more sensitive than RT-qPCR, highlighting its efficiency. Using crude extracts, the sensitivity of both RT-qPCR and RT-ddPCR was reduced. However, the use of crude extract, with the appropriate consideration of the scope of analysis, is valid, as it is faster and cheaper. 

The one-step RT-ddPCR test that emerged from this study is not only repeatable and reproducible but also versatile in its applications. It could prove to be a valuable tool for detecting and quantifying PPV in symptomatic and asymptomatic leaves, as well as in unique matrices such as wood and senescent, lyophilized, and *in vitro* cultivated leaves and shoots. RT-ddPCR’s potential for absolute quantification of the analyzed material is particularly noteworthy, as it allows for a bypass of variability or lack of standard reference material. To our knowledge, this is the first one-step RT-ddPCR assay developed for detecting and quantifying PPV. Its versatility and accuracy demonstrate that this method can be profitably used in diagnostic and certification programs and in assessing the PPV resistance of new elite varieties.

## Figures and Tables

**Table 1:** List of viruses and viroids used in this study.

Viral/Viroid Isolates			
Species	Strain/Isolate	Code	Original Host
Plum pox virus	M	CREA-DC-PPV6	*Prunus persica*
Plum pox virus	D	CREA-DC-PPV7	*Prunus salicina*
Plum pox virus	D	CREA-DC-PPV8	*Prunus domestica*
Plum pox virus	D	CREA-DC-PPV9	*Prunus domestica*
Plum pox virus	D	CREA-DC-PPV10	*Prunus domestica*
Plum pox virus	D	CREA-DC-PPV11	*Prunus salicina*
Plum pox virus	M	CREA-DC-PPV ISPAVE-11	*Prunus persica*
Plum pox virus	D	CREA-DC-PPV ISPAVE-17	*Prunus armeniaca*
Plum pox virus	M	CREA-DC-PPV ISPAVE-44	*Prunus persica*
Plum pox virus	Rec	CREA-DC-PPV Rec BR	*Prunus armeniaca*
Plum pox virus	C/SwC	CREA-DC-PPV SwC	*Prunus avium*
Plum pox virus	EA	CREA-DC-PPV EA	*Prunus armeniaca*
Apple mosaic virus (ApMV)	114	CREA-DC 0807	*Prunus persica*
Apple mosaic virus (ApMV)	Bior	Bior-ApMV	*Humulus lupulus*
Apple chlorotic leafspot virus (ACLSV)	ISF	CREA-DC 07	*Prunus persica*
Prune dwarf virus (PDV)	12	CAV 12	*Prunus persica*
Prunus necrotic ring spot virus (PNRSV)	E	CAV 2022	*Prunus persica*
Prunus necrotic ring spot virus (PNRSV)	1/12	Bior-PNRSV	*Prunus domestica*
Apple stem pitting/ apple stem grooving virus (ASPV/ASGV)	M135	21VIR/22	*Malus domestica*
Peach latent viroid (PLMVd)	C15	CAV 1021	*Prunus persica*
Hop stunt viroid (HSVd)	CDC1	CREA-DC HSVd 1	*Citrus* spp.

PPV strains: M = Marcus; D = Dideron; Rec = Recombinant; C = Cherry (SwC = sweet cherry isolate); EA = El Amar.

**Table 2:** Analytical sensitivity of the RT-qPCR and RT-ddPCR assays for PPV-Rec, PPV-M, and PPV-D strains. For each extraction method, the last dilutions up to the LOD of the RT-ddPCR are reported, showing the mean and standard deviation of Cq and the RT-ddPCR copy number. The copy number in RT-qPCR was calculated using linear regression from RT-ddPCR quantification.

Extraction Method	Isolate	RNA Dilution	RT-qPCR Cq ± SD	PPV RNA Copy Number ± SD (in 1 µL RNA Sample)	
				RT-qPCR	RT-ddPCR
TRNA	CREA-DC-PPV Rec BR	10^−5^	31.3 ± 0.0	74.2 ± 1.8	64 ± 0.3
		10^−6^	Negative ^a^	-	10.5 ± 0.2
		10^−7^	Negative	-	0 ^c^
	CREA-DC-PPV6	10^−5^	31.5 ± 0.2	45.4 ± 7.9	43 ± 0.8
		10^−6^	Negative	-	4.4 ± 0.2
		10^−7^	Negative	-	0 ^c^
	CREA-DC-PPV10	10^−5^	31.9 ± 0.2	38.8 ± 4.8	32.5 ± 1.3
		10^−6^	Negative ^b^	-	3.8 ± 0.0
		10^−7^	Negative	-	0
Crude extract	CREA-DC-PPV Rec BR	10^−3^	33.8 ± 0.7	5.4 ± 3	5 ± 0.1
		10^−4^	Negative ^a^	-	0
	CREA-DC-PPV6	10^−3^	35.2 ± 1.1	6.0 ± 3.7	5.9 ± 0.2
		10^−4^	Negative ^b^	-	0
	CREA-DC-PPV10	10^−2^	32.4 ± 0.4	15.5 ± 4.4	16.0 ± 0.0
		10^−3^	36.4 ± 0.9	1.0 ± 0.5	0 ^c^
		10^−4^	Negative	-	0

SD—standard deviation (n = 3 for RT-qPCR; n = 2 for RT-ddPCR). ^a^ Signal was observed in two of three replicates; curves were flattened. ^b^ Signal was observed in one of three replicates; curves were flattened. ^c^ Signal with one or two positive droplets, no accurate target copy calculation.

**Table 3:** Results of the selectivity test obtained by spiking CREA-DC-PPV 6 TRNA in seven matrices.

Matrixes Spiked with PPV-D	RT-qPCR Cq ± SD	RT-ddPCR RNA Copy Number/µL ± SD (in 1 µL RNA Sample)
H_2_O	17.8 ± 0.2	2415 ± 2.1
Peach (*P. persica*)	17.9 ± 0.2	2400 ± 5.7
Apricot (*P. armeniaca*)	17.8 ± 0.2	2515 ± 7.8
Plum (*P. domestica*)	17.6 ± 0.1	2355 ± 2.1
Japanese plum (*P. salicina*)	17.6 ± 0.1	2590 ± 9.9
Sweet cherry (*P. avium*)	17.5 ± 0.1	2545 ± 0.7
Myrobalan plum (*P. cerasifera*)	17.4 ± 0.1	2605 ± 3.5
Almond (*P. amygdalus dulcis*)	17.3 ± 0.1	2540 ± 1.4

SD = standard deviation.

## Data Availability

Publicly available datasets were analyzed in this study. These data can be found here: https://www.ncbi.nlm.nih.gov/nucleotide/, accessed on 20 March 2024. Accession numbers are reported in the “Results and Discussion” section, “Reverse primer design and its application in RT-qPCR” paragraph.

## Supplementary Materials

The following supporting information can be downloaded at: https://www.mdpi.com/article/10.3390/plants13233276/s1. Figure S1: Comparison of the qPCR analysis on PPV obtained using the Olmos set and the set developed in this study. Figure S2: Optimization of the RNA reverse transcription process. Figure S3: Comparison of droplet separation after 40 and 45 amplification cycles. Figure S4: Results of the inclusivity test in RT-qPCR and RT-ddPCR. Figure S5: Detection of PPV in infected GF305 wood, senescent leaves, and asymptomatic leaves. Figure S6: *In vitro* explant of healthy peach ‘GF305’ and plum-pox-virus-infected peach ‘GF305’. Table S1: Number of PPV CP sequences showing different nucleotides compared to the oligos sequences on the total number of sequences analyzed for each PPV strain. Table S2: Specificity tests *in vitro* and *in vivo* based on viruses occurring in the EPPO region. Table S3: Experimental results of RT-qPCR for the evaluation of repeatability and reproducibility criteria. Table S4: Experimental results of RT-ddPCR for the evaluation of repeatability and reproducibility criteria.
