# Supplementary material for: Development and Validation of One-Step Reverse Transcription-Droplet Digital PCR for Plum Pox Virus Detection and Quantification from Plant Purified RNA and Crude Extract
Source: Plants (Basel). 2024 Nov 22;13(23):3276. doi: 10.3390/plants13233276 (PMC11644555; doi:10.3390/plants13233276)
Supplement: Supplementary file 1 [file plants-13-03276-s001.zip › Supplementary Figure S1 Bertinelli et al.pdf]

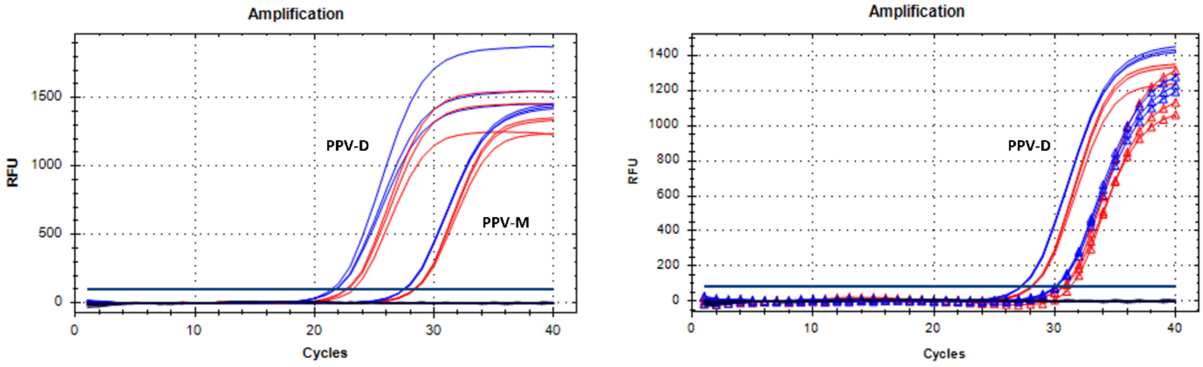

(a) (b)

Supplementary Figure S1. Comparison of the qPCR analysis on PPV obtained using the Olmos set (Olmos et al., 2005; red curves) and the set developed in this study (blue curves). (**a**) TRNAs of CREA-DC-PPV7 (PPV-D) and CREA-DC-PPV6 (PPV-M); (**b**) comparison of results obtained using TRNAs (simple curves) and crude extracts (triangles) of CREA-DC-PPV6. Each sample was tested in triplicate. For a better view of the results only two and one PPV isolates are shown, respectively.
