# Supplementary material for: Development and Validation of One-Step Reverse Transcription-Droplet Digital PCR for Plum Pox Virus Detection and Quantification from Plant Purified RNA and Crude Extract
Source: Plants (Basel). 2024 Nov 22;13(23):3276. doi: 10.3390/plants13233276 (PMC11644555; doi:10.3390/plants13233276)
Supplement: Supplementary file 1 [file plants-13-03276-s001.zip › Supplementary Figure S2 Bertinelli et al.pdf]

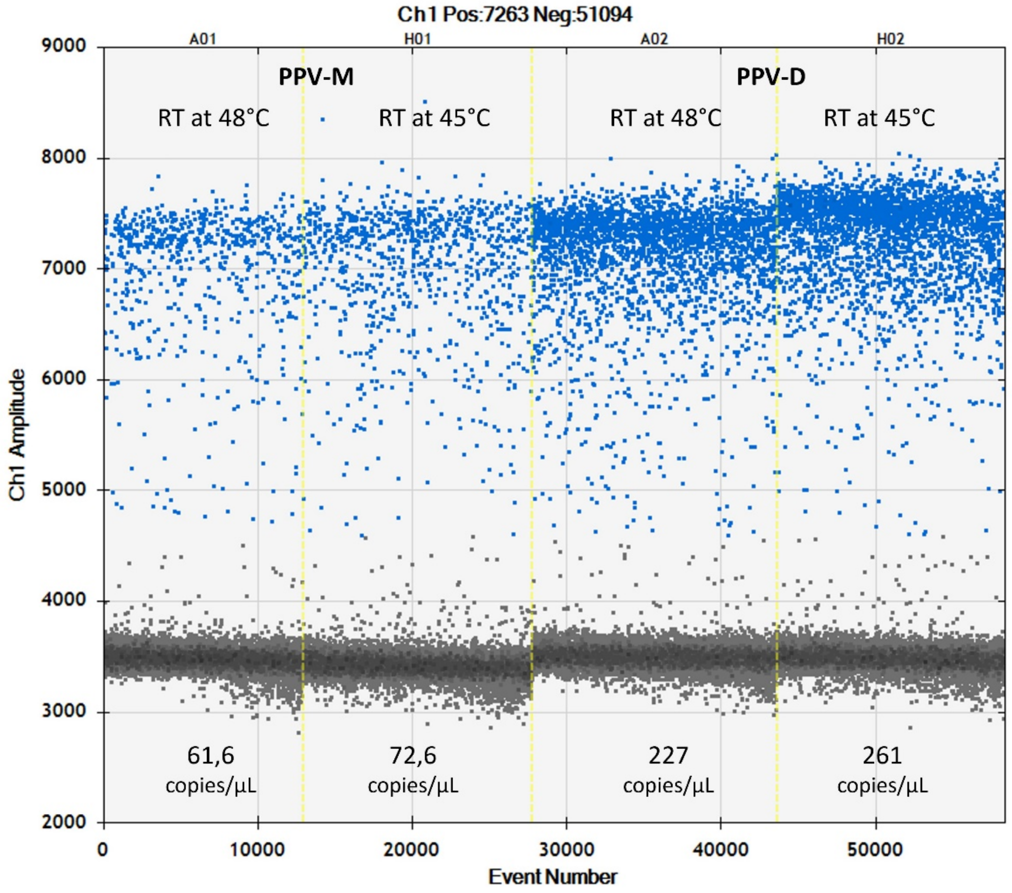

Supplementary Figure S2. Optimization of RNA reverse transcription process. The figure shows the TRNAs of CREA-DC-PPV6 (PPV-M strain, left side) and CREA-DC-PPV7 (PPV-D strain, right side) both tested comparing retrotranscription step (RT) of 48°C and 45 °C.
