# Supplementary material for: Development and Validation of One-Step Reverse Transcription-Droplet Digital PCR for Plum Pox Virus Detection and Quantification from Plant Purified RNA and Crude Extract
Source: Plants (Basel). 2024 Nov 22;13(23):3276. doi: 10.3390/plants13233276 (PMC11644555; doi:10.3390/plants13233276)
Supplement: Supplementary file 1 [file plants-13-03276-s001.zip › Supplementary Figure S3 Bertinelli et al .pdf]

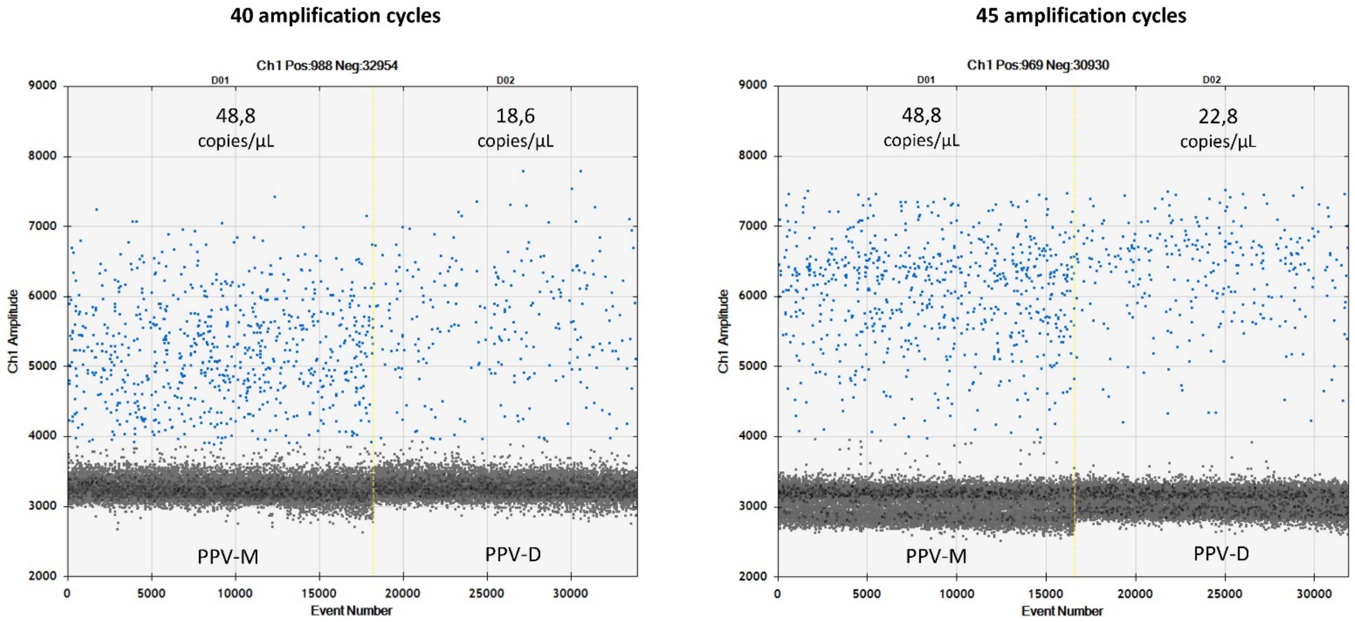

Supplementary Figure S3. Comparison of droplet separation after 40 (left panel) and 45 (right panel) amplification cycles. Figure shows the amplification of TRNAs of CREA-DC-PPV6 (PPV-M strain) and CREA-DC-PPV11 (PPV-D strain)
