# Supplementary material for: Development and Validation of One-Step Reverse Transcription-Droplet Digital PCR for Plum Pox Virus Detection and Quantification from Plant Purified RNA and Crude Extract
Source: Plants (Basel). 2024 Nov 22;13(23):3276. doi: 10.3390/plants13233276 (PMC11644555; doi:10.3390/plants13233276)
Supplement: Supplementary file 1 [file plants-13-03276-s001.zip › Supplementary Figure S4 Bertinelli et al.pdf]

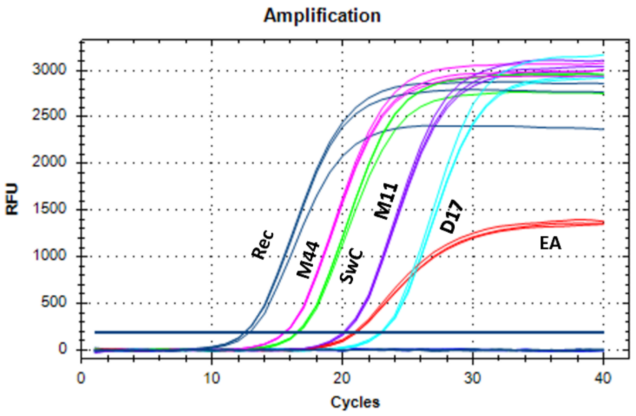

(**a**)

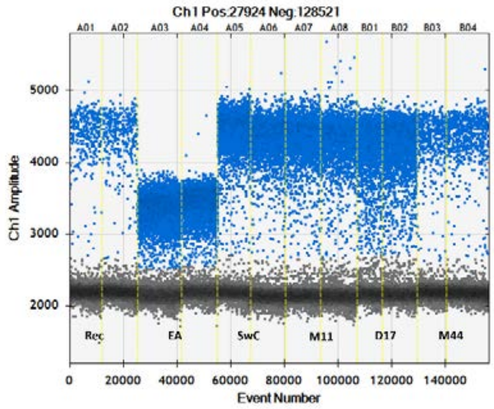

(**b**)

Supplementary Figure S4. Results of the inclusivity test in RT-qPCR and RT-ddPCR for five PPV strains (strains are reported in figure). **(a)** TRNAs tested in triplicate in RT-qPCR; (**b**) TRNAs tested in duplicate in RT-ddPCR.
