# Supplementary material for: Development and Validation of One-Step Reverse Transcription-Droplet Digital PCR for Plum Pox Virus Detection and Quantification from Plant Purified RNA and Crude Extract
Source: Plants (Basel). 2024 Nov 22;13(23):3276. doi: 10.3390/plants13233276 (PMC11644555; doi:10.3390/plants13233276)
Supplement: Supplementary file 1 [file plants-13-03276-s001.zip › Supplementary Figure S5 Bertinelli et al.pdf]

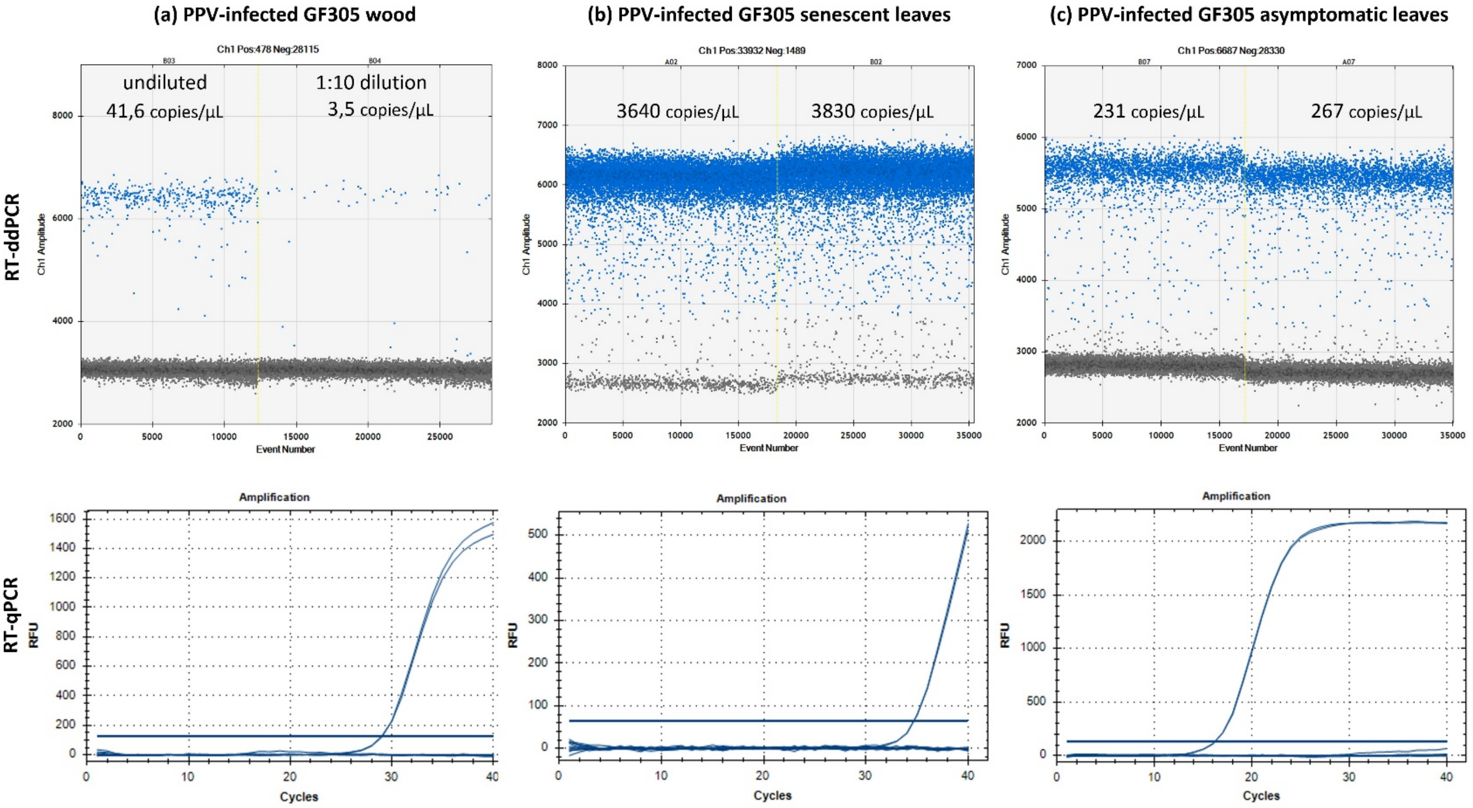

Supplementary Figure S5. Detection of PPV in infected GF305 wood, senescent leaves, and asymptomatic leaves

(a) **RT-ddPCR.** CREA-DC-PPV10 (PPV-D) infected GF305 wood sample (undiluted and 1:10 dilution)
RT-qPCR. CREA-DC-PPV10 (PPV-D) infected GF305 wood sample (undiluted technical duplicate)

(b) **RT-ddPCR** CREA-DC-PPV6 (PPV-M) infected GF305 senescent leaves (undiluted technical duplicate)
RT-qPCR (CREA-DC-PPV6 (PPV-M) infected GF305 senescent leaves (undiluted technical duplicate)

(c) **RT-ddPCR** CREA-DC-PPV11 (PPV-D) infected GF305 asymptomatic leaves (undiluted technical duplicate)
RT-qPCR CREA-DC-PPV11 (PPV-D) infected GF305 asymptomatic leaves (undiluted technical duplicate)
