# Supplementary material for: Development and Validation of One-Step Reverse Transcription-Droplet Digital PCR for Plum Pox Virus Detection and Quantification from Plant Purified RNA and Crude Extract
Source: Plants (Basel). 2024 Nov 22;13(23):3276. doi: 10.3390/plants13233276 (PMC11644555; doi:10.3390/plants13233276)
Supplement: Supplementary file 1 [file plants-13-03276-s001.zip › Supplementary Figure S6 Bertinelli et al.pdf]

a) b)

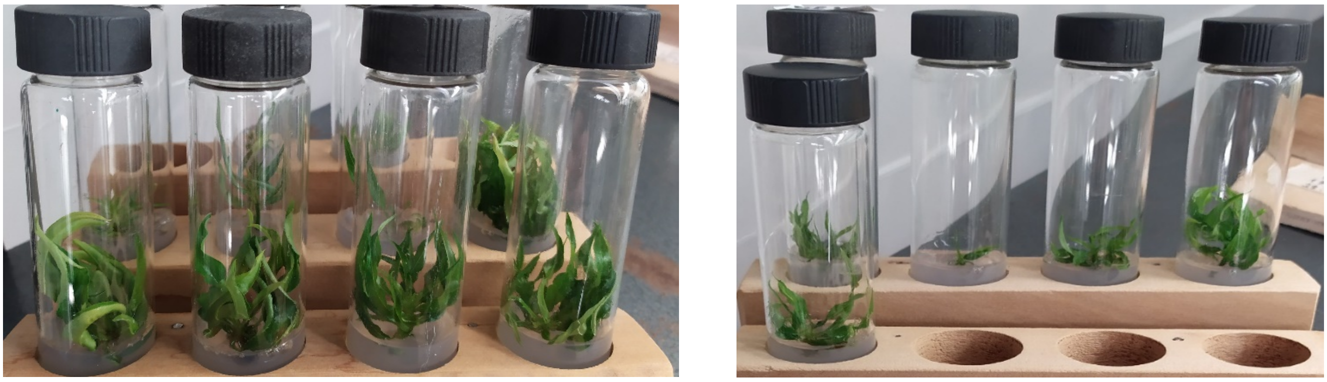

Supplementary Figure 6. *In vitro* explants of a) healthy peach 'GF305'; b) plum pox virus-infected peach 'GF305'
