# Supplementary material for: Development and Validation of One-Step Reverse Transcription-Droplet Digital PCR for Plum Pox Virus Detection and Quantification from Plant Purified RNA and Crude Extract
Source: Plants (Basel). 2024 Nov 22;13(23):3276. doi: 10.3390/plants13233276 (PMC11644555; doi:10.3390/plants13233276)
Supplement: Supplementary file 1 [file plants-13-03276-s001.zip › Supplementary Table S1 Bertinelli et al.pdf]

Oligo	PPV strains	n. of sequences with difference compared to the oligo sequence/n. of total PPV *CP* sequences analyzed							
		PPV-D	PPV-M	PPV-Rec	PPV-T	PPV-EA	PPV-C	PPV-SC	PPV-W
	P241-F	2/315	4/68	0/17	5/56	0/2	0/11	1/10	5/6[2]
	PPVrUn-R	4/315	2/68	0/17	0/56	0/2	0/11	0/10	6/6[1]
	Probe PPV-DM	6/315	2/68	0/17	3/56	2/2[1]	0/11	8/10[1]	6/6[1]

Supplementary Table S1. Number of PPV CP sequences showing different nucleotides compared to the oligos sequences on the total number of sequences analyzed for each PPV strain. Nucleotide variations of the *CP* target region were evidenced by the clustal Omega tool. Differences consisted of 1 to a maximum of 3 different nucleotides per oligo. Most of the differences were found in internal regions. For EA, CR, and W, one nucleotide was substituted in the same position.

[1] substitution of C with T
[2] substitution of T with C
