# Supplementary material for: Development and Validation of One-Step Reverse Transcription-Droplet Digital PCR for Plum Pox Virus Detection and Quantification from Plant Purified RNA and Crude Extract
Source: Plants (Basel). 2024 Nov 22;13(23):3276. doi: 10.3390/plants13233276 (PMC11644555; doi:10.3390/plants13233276)
Supplement: Supplementary file 1 [file plants-13-03276-s001.zip › Supplementary Table S2 Bertinelli et al.pdf]

**Virus name**	*in silico* analysis	*in vivo* analysis	**Occurrence in the EPPO region**
Apple mosaic virus (ApMV)	-	-	✓
Apple chlorotic leafspot virus (ACLSV)	-	-	✓
Prune dwarf virus (PDV)	-	-	✓
Prunus necrotic ring spot virus (PNRSV)	-	-	✓
Cherry mottle leaf virus (CMLV)	-	nt	
Cherry green ring mottle virus (CGRMV)	-	nt	
Little cherry virus 1 (LChV 1)	-	nt	
Little cherry virus 2 (LChV 2)	-	nt	
Apricot latent virus (ApLV)	-	nt	
Strawberry latent ringspot virus (SLRSV)	-	nt	
Apple stem pitting/Apple stem grooving virus (ASPV/ASGV)	nt	-	
Peach latent viroid (PLVd)	nt	-	✓
Hop stunt viroid (HSVd)	nt	-	
PPV-M ISPAVE-44	nt	+	✓
PPV-D ISPAVE-17	nt	+	✓
PPV-M ISPAVE-11	nt	+	✓
PPV-Rec BR	nt	+	
PPV-SwC	nt	+	
PPV-EA	nt	+	

Supplementary Table S2. List of viruses and viroids infecting almond, apricot, peach and plum considered in the specificity assays evaluated *in silico* and/or *in vitro* (by both RT-qPCR and RT-ddPCR). –, no matches found; +, matches found; nt, not tested; ✓Viruses reported by PM 4/30(1) occurring in the EPPO region. (a), tests performed using TRNA extracted by kit; (b), tests performed using crude extract.
