# Supplementary material for: Development and Validation of One-Step Reverse Transcription-Droplet Digital PCR for Plum Pox Virus Detection and Quantification from Plant Purified RNA and Crude Extract
Source: Plants (Basel). 2024 Nov 22;13(23):3276. doi: 10.3390/plants13233276 (PMC11644555; doi:10.3390/plants13233276)
Supplement: Supplementary file 1 [file plants-13-03276-s001.zip › Supplementary Table S3 RT-qPCR repeatability reproducibility.pdf]

Performance Criterion	Template	PPV Isolate	Sample dilution	Agreement among replicates	RT-qPCR Cq ± SD
Repeatability [a]	TRNA	CREA-DC-PPV Rec BR	10^{-4}	100 %	28.1 ± 0.1
		CREA-DC-PPV 6	10^{-4}	100 %	28.3 ± 0.1
		CREA-DC-PPV 10	10^{-4}	100 %	29.1 ± 0.1
	Crude extracts	CREA-DC-PPV Rec BR	10^{-1}	100 %	27.1 ± 0.5
		CREA-DC-PPV 6	10^{-1}	100 %	27.1 ± 0.0
		CREA-DC-PPV 10	10^{-1}	100 %	29.4 ± 0.2
Reproducibility [b]	TRNA	CREA-DC-PPV Rec BR	10^{-4}	100 %	29.4 ± 0.1
		CREA-DC-PPV 6	10^{-4}	100 %	29.7 ± 0.1
		CREA-DC-PPV 10	10^{-4}	100 %	30.3 ± 0.1
	Crude extracts	CREA-DC-PPV Rec BR	10^{-1}	100 %	28.7 ± 0.3
		CREA-DC-PPV 6	10^{-1}	100 %	28.6 ± 0.4
		CREA-DC-PPV 10	10^{-1}	100 %	31.3 ± 0.7

Supplementary Table S3. Experimental results of RT-qPCR for the evaluation of repeatability and reproducibility criteria performed on CREA-DC-PPV Rec BR (PPV-REC = PPV Recombinant strain), CREA-DC-PPV10 (PPV-D = Dideron strain) and CREA-DC-PPV6 (PPV-M = PPV Marcus strain) TRNAs and crude extracts.

a) Repeatability: three replicates of sample extracts at low concentrations were analyzed for each PPV isolate (Cq mean and standard deviation are shown).

b) Reproducibility: as for repeatability, but with two different operators, on different days and with different equipment.
