# Supplementary material for: Development and Validation of One-Step Reverse Transcription-Droplet Digital PCR for Plum Pox Virus Detection and Quantification from Plant Purified RNA and Crude Extract
Source: Plants (Basel). 2024 Nov 22;13(23):3276. doi: 10.3390/plants13233276 (PMC11644555; doi:10.3390/plants13233276)
Supplement: Supplementary file 1 [file plants-13-03276-s001.zip › Supplementary Table S4 RT-ddPCR repeatability reproducibility.pdf]

Performance Criterion	Template	PPV Isolate	Sample dilution	Agreement among replicates	PPV RNA copy number ± SD (in 1 μL RNA sample)
Repeatability [a]	TRNA	CREA-DC-PPV Rec BR	10^{-4}	100 %	467.7 ± 10.5
		CREA-DC-PPV 6	10^{-4}	100 %	436.3 ± 4.1
		CREA-DC-PPV 10	10^{-4}	100 %	231.7 ± 1.7
	Crude extracts	CREA-DC-PPV Rec BR	10^{-2}	100 %	32.3 ± 0.6
		CREA-DC-PPV 6	10^{-2}	100 %	76.0 ± 1.7
		CREA-DC-PPV 10	10^{-2}	100 %	17.5 ± 0.8
Reproducibility [b]	TRNA	CREA-DC-PPV Rec BR	10^{-4}	100 %	489.3 ± 1.4
		CREA-DC-PPV 6	10^{-4}	100 %	342.7 ± 4.9
		CREA-DC-PPV 10	10^{-4}	100 %	248.7 ± 2.7
	Crude extracts	CREA-DC-PPV Rec BR	10^{-2}	100 %	39.7 ± 0.3
		CREA-DC-PPV 6	10^{-2}	100 %	78.0 ± 1
		CREA-DC-PPV 10	10^{-2}	100 %	12.3 ± 0.1

Supplementary Table S4. Experimental results of RT-ddPCR for the evaluation of repeatability and reproducibility criteria performed on CREA-DC-PPV Rec BR (PPV-REC = PPV Recombinant strain), CREA-DC-PPV10 (PPV-D = Dideron strain) and CREA-DC-PPV6 (PPV-M = PPV Marcus strain) TRNAs and crude extracts.

a) Repeatability: three replicates of sample extracts at low concentrations were analyzed for each PPV isolate (RNA copy number mean and standard deviation are shown).

b) Reproducibility: as for repeatability, but with two different operators, on different days, and with different equipment.
